# Systematic selection of suitable reference genes for quantitative real-time PCR normalization studies of gene expression in *Lutjanus* *erythropterus*

**DOI:** 10.1038/s41598-024-63335-x

**Published:** 2024-06-10

**Authors:** Lujun Chen, Qiulu Liang, Zhuoxin Lai, Haitao Cui, Zhenmin Xu, Zizhao Chen, Zhongdian Dong, Zhongduo Wang, Yusong Guo

**Affiliations:** 1 Key Laboratory of Aquaculture in South China Sea for Aquatic Economic Animal of Guangdong Higher Education Institutes, Fisheries College, Guangdong Ocean University, Zhanjiang, 524025 China; 2 Guangdong Provincial Key Laboratory of Aquatic Animal Disease Control and Healthy Culture, Fisheries College, Guangdong Ocean University, Zhanjiang, 524088 China

**Keywords:** *Lutjanuserythropterus*, Reference gene, qRT-PCR, Transcriptome, Molecular biology, Zoology, Ocean sciences

## Abstract

Quantitative real-time PCR (qRT-PCR) has been widely employed for the study of gene expression in fish, and accurate normalization is crucial. In this study, we aimed to identify the most stably expressed genes in various tissues, different developmental stages, and within astaxanthin treatment groups in *Lutjanus* *erythropterus*. Twelve candidate genes (*EEF1A*, *CYB5R3*, *DLD*, *IDH3A*, *MRPL17*, *MRPL43*, *NDUFS7*, *PABPC1*, *PAGR1*, *PFDN2*, *PSMC3*, and *RAB10*) were examined via qRT-PCR. We employed geNorm and NormFinder to assess their stability. The results revealed that *RAB10* and *PFDN2* exhibited relatively stable expression patterns across different tissue and astaxanthin treatment groups, while *NDUFS7* and *MRPL17* proved to be the most reliable reference gene combinations across various developmental stages. The stability of these selected genes was further validated by assessing the expression of two target genes, *CRADD* and *CAPNS1*, across developmental stages, reinforcing the reliability of NDUFS7 as it closely aligned with transcriptome-wide expression patterns at these stages. The present results will help researchers to obtain more accurate results in future qRT-PCR analysis in *L.* *erythropterus.*

## Introduction

At present, leveraging high-throughput transcriptome sequencing, transcriptome analysis, and data mining has become an effective means to screen differentially expressed genes and quantify the expression abundance of their transcripts. qRT-PCR has emerged as a powerful tool for verifying gene expression profiles due to its high sensitivity, specificity, good reproducibility, simple operation, and other advantages^1,2^. Ideally, reference genes should exhibit consistent expression levels across a spectrum of developmental stages, diverse tissue types, and various physiological conditions^3,4^. However, there is clear evidence that no single reference gene can maintain a constant level of expression in different fish species, tissues, or experimental conditions^5–8^. Therefore, it is necessary to screen several stably expressed reference genes before normalizing the expression levels of target genes based on qRT-PCR.

Crimson snapper (*Lutjanus* *erythropterus*) belongs to the subtropical and middle-lower tropical fish of the class Actinopterygii, order Perciformes, family Lutjanidae, and genus *Lutjanus*. It is primarily distributed in the Indo-West Pacific, the Indian Ocean, and the South China Sea (https://www.fishbase.se). *L.* *erythropterus* is an economically significant marine fish known for its delectable flesh and its richness in protein and other nutritional values^9^. Its characteristic red hue, a vital aesthetic and health marker, is intimately linked to traits such as growth rate and stress resilience, yet frequently undergoes undesirable alterations during farming, leading to substantial financial losses^10^. To counteract this, cultivators often administer astaxanthin supplements or include carotenoid-rich diets like shrimp heads in the weeks preceding harvest to enhance color vibrancy. Astaxanthin, a potent natural carotenoid abundant in aquatic life, flora, algae, and fungi, is a ubiquitous color intensifier in aquaculture practices^11–13^. Elucidating the genetic underpinnings of growth and pigmentation in *L.* *erythropterus* necessitates the examination of gene expression dynamics under varying conditions. Quantitative real-time polymerase chain reaction (qRT-PCR) stands as a cornerstone in gene expression analysis, with the choice of internal reference genes being pivotal for accurate qRT-PCR outcomes. In recent years, molecular-level studies on *L.* *erythropterus* have primarily focused on molecular genetic markers^9,14^ and functional genes related to growth and development^15,16^, while research on the screening of reference genes has not been reported.

The aim of this study was to screen for suitable reference genes in different tissues, at different developmental stages, and under astaxanthin treatment groups. Non-differential gene in the crossover were selected by data from three transcriptomes of *L.* *erythropterus*, and then 12 of these genes with high and stable expression were chosen as candidate internal reference genes. The stability of 12 candidate genes (*EEF1A*, *CYB5R3*, *DLD*, *NAD(*+*)*, *IDH3A*, *MRPL17*, *MRPL43*, *NDUFS7*, *PABPC1*, *PAGR1*, *PFDN2*, *PSMC3*, *and RAB10*) was analyzed using NormFinder and geNorm software. The relatively stable internal reference genes were selected, providing a theoretical basis for identifying the best endogenous genes and establishing a foundation for the analysis of related genes in *L.* *erythropterus.*

## Materials and methods

### Animals

Ten different developmental stages of *L.* *erythropterus* were collected from Hainan Blue Granary Technology Co. Ltd for the experiment. For developmental stages blastopore closure (Bc), optic chorionic (Oc), eye formation (Elf), heart beating (Hb), and hatching (Hat), with 30–40 fertilised eggs sampled per tube and three tubes were collected. Furthermore, five larval stages were sampled at 1, 3, 10, 15, and 2 days after hatching with one sampled per tube and three tubes were collected. For different tissues, we collected samples from three adult fishes for the experiment. The experimental adult fish were obtained from the Zhanjiang Xia Shan Aquatic Products Wholesale Market (Guangdong, China). The fish underwent anesthesia and euthanasia using MS-222 (Sigma, USA). Ten pieces of tissues were collected from each, including the liver, intestine, muscle, lateral skin, dorsal skin, abdominal skin, spleen, heart, retina, and brain.

The juvenile of *L. erythropterus* (average initial body length, 4.81 ± 0.32 cm, average initial body weight, 3.29 ± 0.17 g) used in the experiment were obtained from Yangjiang Fishery Township Aquatic Science and Technology Industrial Co. Ltd. The experiment was conducted at the East Sea Island Aquaculture Base of Guangdong Ocean University (Guangdong, China). Initially, the juvenile snappers were placed in rearing tanks measuring 4 × 3 × 1.5 m, with a salinity level of 32 ± 1.93 ppt, a water temperature of 30.5 ± 0.69 °C, pH levels exceeding 7.8, and dissolved oxygen levels exceeding 6.9 mg/L. They were temporarily raised in these conditions for 7 days to acclimate. Subsequently, healthy *L.* *erythropterus* of consistent size were randomly assigned to the control group and the treatment group. The control group was fed a basic diet without the addition of astaxanthin, while the treatment group was fed a diet enriched with 200 mg/kg astaxanthin. They were fed twice daily, once in the morning at 8:00 and once in the afternoon at 4:00, with each feeding aimed at ensuring satiation. This feeding regimen continued into the 4 and 6 weeks of the experiment. For sampling, the fish were anesthetized using MS-222 (Sigma, USA) three fish were randomly selected from each group, and a total of nine fish skin tissues were randomly taken from the experimental group and the control group, each transcriptome sample has three biological replicates. These samples were rapidly frozen using liquid nitrogen and stored at − 80 ℃ for subsequent experiments. All experimental protocols were approved by the Animal Research and Ethics Committees of the Institute of Aquatic Economic Animals of Guangdong Ocean University, Zhanjiang, Guangdong, China (201903003). The study does not involve endangered or protected species. Further, all study protocols were performed according to the Explanation and Elaboration accordance to the ARRIVE guidelines 2.0. All methods were performed following relevant guidelines and regulations.

### RNA extraction and cDNA synthesis

Total RNA was extracted from sample using Trizol reagent (Invitrogen, USA) according to the manufacturer’s instructions. The extracted RNA was resuspended in diethylpyrocarbonate (DEPC)-treated water and quantified using a Nanodrop 2000 (Thermo, USA) spectrophotometer at 260 nm and 280 nm. RNA integrity was analyzed via electrophoresis on a 1% agarose gel. The RNA samples were stored at − 80 ℃. First-strand cDNA was synthesized from each total RNA sample using the HiScript@ II reverse transcriptase kit (vazyme) according to the manufacturer’s protocol.

### Candidate reference genes selection and primer design

To select new candidate reference genes, we analyzed the transcriptome sequencing data from three different transcriptomes including different tissues (transcriptome data from different tissues was collected from our previous studies^15^), developmental stages (accession no. PRJNA946949), and astaxanthin-treated groups (accession no. PRJNA1039708) in the *L.* *erythropterus*. We identified non-differentially expressed genes in the three transcriptomes, with 2959, 2959, and 92012 non-differentially expressed genes in different tissues, different developmental stages, and after astaxanthin treatment groups, respectively. Then, the intersection of the three sets of data was taken, with the condition that the gene expression was not zero, and a total of 68 co-expressed genes were obtained. We sorted the expression levels of these 68 co-expressed genes in the blood tissue and selected the top 12 genes with the highest expression levels as candidate reference genes for this study for experiments. Primers for the *RAB10*, *PFDN2*, *PABPC1*, *IDH3A*, *PAGR1*, *DLD*, *EEF1A*, *CYB5R3*, *MRPL17*, *PSMC3*, *MRPL43*, and *NDUFS7* genes were designed using NCBI Primer-BLAST.

### qRT-PCR assay

A mixture of cDNA from different tissues of *L.* *erythropterus* was used as templates, and eight cDNAs of different concentrations were obtained by successive four-fold gradient dilutions, and used as templates. qRT-PCR was performed using the LightCycler^®^ 96 PCR system (Roche, Basel, Switzerland). Each sample comprised three biological replicates and two technical replicates. The final amplification volume was 10.0 μL, consisting of 0.5 μL cDNA (10 μM), 5.0 μL 2× SYBR^®^ Green qPCR mix, 0.2 μL of each primer (10 μM), and 4.1 μL ddH_2_O. PCR amplification was carried out in duplicate, and a no-cDNA reaction was used as a negative control. The reaction conditions were as follows: 94 ℃ for 180 s, followed by 40 cycles of 94 ℃ for 15 s, 60 ℃ for 15 s, and 72 ℃ for 20 s. Melting curves of candidate internal reference genes were analysed using LightCycler^®^ 96 instrumentation software to verify primer specificity. Based on the Ct values, a standard curve was constructed, and the slope k and linear correlation coefficient R^2^ were calculated. Finally, the amplification efficiency E (E = 10^− 1/k^ − 1) of the reference gene was determined^17^. According to the results of the standard curve, all sample cDNAs were diluted ten-fold, and qRT-PCR amplification was performed for all genes, with the same reaction system and conditions as above.

### Stability expression analysis

The abundance of the selected candidate reference genes in different samples was analyzed through a direct comparison of Ct values. Among the 12 selected reference genes, we determined the most stable reference genes using three widely used methods: BestKeeper, NormFinder, and geNorm. Subsequently, the candidate reference genes were comprehensively ranked using RefFinder (https://blooge.cn/RefFinder/). BestKeeper assessed the expression stability of the endogenous genes directly based on the standard deviation (SD) of the CT values^18^. In the case of geNorm and NormFinder, the amplified Ct values of the samples need to be converted into Q-values and then imported into the software for stability analysis^19,20^. The Q value is calculated as follows: Q = E(Ct min − Ct sample), where E represents the amplification efficiency of the gene, Ct min is the minimum amplified Ct value among the candidate internal reference genes across different sample series, and Ct sample is the amplification Ct value in different sample series for the candidate internal reference gene. When the amplification efficiency is close to 100%, E is set to 2, and the maximum relative number for each gene is normalized to 1. NormFinder introduces an ANOV-directed model to calculate intra- and inter-group variation in Q-values and to rank the internal reference genes according to stability values (SV). geNorm determines the gene expression stability index, M, by calculating the average paired-variance value of the internal reference genes with respect to all other genes included in the same analysis. Additionally, geNorm calculates the pairwise differences of the internal reference genes, denoted as V_n/n+1_, and determines the optimal selection of the number of internal reference genes in qRT-PCR experiments based on these V_n/n+1_ values.

### Validation of the stability of reference genes

To further validate the stability of the candidate internal reference genes, we selected two genes each that were the most stable and the least stable during different developmental stages to serve as reference genes, based on the analysis of reference gene stability across development. Within the intersection of differentially expressed genes from three transcriptomes of *L.* *erythropterus*, including different tissues, different developmental stages, and astaxanthin-treated groups, two genes, *CRADD* (CASPsase-2 and RIPK1 domain-containing adapter with a death domain) and *CAPNS1* (calpain small subunit 1), were randomly selected as target genes for qRT-PCR analysis. This analysis entailed three biological replicates and three technical replicates. According to the qRT-PCR primer design principles, primers for the target genes were designed using Primer Premier 5.0 (see Supplementary Table S1). The relative expression levels of target genes under different reference genes were calculated using the 2^−ΔΔCt^ method and compared with the differential expression outcomes from the transcriptomic data to assess the consistency between qRT-PCR and transcriptomic datasets.

## Results

### Analysis of primer specificity and amplification efficiency

Calculated from the results of the standard curves (see Supplementary Fig. S1), the polymerase chain reaction efficiency (E) of 12 candidate genes ranged from 90 to 109%, and the correlation coefficient (R^2^) ranged from 0.98 to 1.0, indicating that the candidate internal reference genes exhibited good amplification efficiency (Table 1). Additionally, melting curve analysis showed that all primers produced a major peak, confirming their amplification specificity (see Supplementary Fig. S2), thus indicating that the 12 primer pairs exhibited good specificity.

**Table 1:** Primers and PCR efficiencies of candidate reference genes.

Gene	Primer (5ʹ-3ʹ)	Product size (bp)	Efficiency (%)	Correlation (R^2^)
*EEF1A*	F: TGTCACCTTCGCTCCTCC R: GGTCGTTCTTGCTGTCGC	167	98.0	0.9975
*PABPC1*	F: ATACCTCAGGCCCAAAACCG R: GCATAGCGTTGGGCATGTTC	127	90.3	0.9992
*NDUFS7*	F: CCAGCTCTGCGAAAGGTGTA R: TTCGGTCACAACCTCTGACG	124	102.5	0.9958
*RAB10*	F: GAGGGTCGTACCAAAAGCCA R: GTTGGCCTTAGCACTCGTCT	82	104.8	0.9958
*IDH3A*	F: GACCTTCGACCTTTACGCCA R: TTCGATGCCGCTGTACTCTC	124	97.7	0.9954
*PAGR1*	F: GCTCCCATACAGTGACGAGG R: GCATCTCCCCTTTAGCGAGG	101	102.1	0.9959
*PSMC3*	F: CACAGAGCAGTACAGCGACA R: AGGTGGTCCGTACATCAGGA	145	96.3	0.9895
*DLD*	F: GGGTGGGACCTGTCTGAATG R: TCCAGGTTCAACGAGATGCC	135	96.5	0.997
*CYB5R3*	F: CGTCAGTCACGACACAAGGA R: CGACCAGCTTCCCGTCTATC	116	102.5	0.9916
*PFDN2*	F: CCGCATTGGAAGCCAACAAA R: GTTCCCGGTACTCTGTGAGC	99	97.2	0.9942
*MRPL43*	F: CAGAACGGTGTCGGTCGTTA R: GAACTCCCTGACTCCCAACG	93	97.5	0.9933
*MRPL17*	F: GTCAAGTGGCTACACACGGA R: GAGAGGTGGGAAGGGATTGC	103	108	0.9981

### Comparative expression levels of candidate reference genes

**Figure 1:**
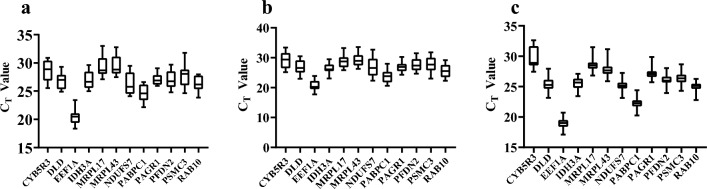
Expression levels of candidate reference genes. (**a**) Astaxanthin treatment groups. (**b**) Different tissues. (**c**) Different developmental stages. The line across the box represents the median, and the whisker caps show the maximum and minimum values.

The Ct value of qRT-PCR directly reflects gene expression abundance, with a smaller Ct value correlating with higher gene expression. We amplified 12 candidate internal reference genes using qRT-PCR from different developmental stages, astaxanthin treatment groups, and adult fish tissues of *L.* *erythropterus*. The distribution of Ct values for the 12 candidate internal reference genes was analyzed after calculating the mean values of the raw Ct values. In the skin tissues after astaxanthin treatment groups, the Ct values ranged from 18.36 to 32.78 (Fig. 1a), with the *EEF1A* gene showing the highest expression (mean value of 20.40) and MRPL43 displaying the lowest expression (mean value of 29.47). In different tissues, the Ct values ranged from 17.73 to 33.59 (Fig. 1b), with *EEF1A* gene exhibiting the highest expression (mean value of 20.73), and *MRPL43* displaying the lowest expression (mean value of 29.27). The data for *PAGR1* showed a more concentrated and less fluctuating range, while *CYB5R3* and *NDUFS7* displayed more fluctuations. For the different developmental stages, the Ct values of the candidate reference genes ranged from 10.12 to 28.35 (Fig. 1c). Among these reference genes, *EEF1A* showed the highest expression level (average Ct = 18.9), while *CYB5R3* exhibited the lowest transcription level (average Ct = 29.7). Overall, the Ct values of the 12 candidate endogenous genes fluctuated widely.

### Stability of expression of candidate genes in *L. erythropterus*

#### BestKeeper analysis

**Figure 2:**
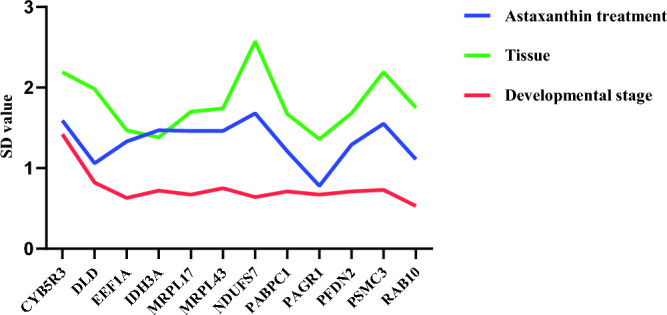
BestKeeper analysis of the stability of candidate internal reference genes. Blue color represents the astaxanthin treatment groups. Green represents different tissues. Red represents different developmental stages.

BestKeeper determines the stability of the reference genes by calculating the standard deviation (SD) and the coefficient of variation (CV). A default threshold 1.0 for the SD value indicates stable expression^18^. In the astaxanthin treatment group, *PAGR1* was the most stable reference gene with an SD value < 1.0, and *RAB10* showed the most stable expression across different developmental stages (SD value = 0.53) (Fig. 2). However, in the analysis of different tissues, all genes had SD values > 1, making BestKeeper unsuitable for selecting reference genes among different tissues.

#### NormFinder analysis

**Figure 3:**
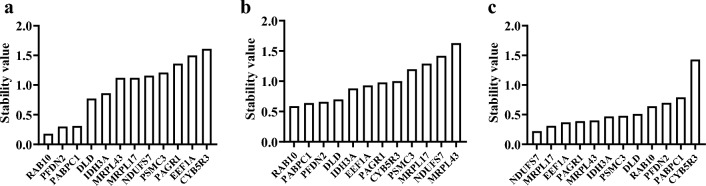
NormFinder analysis of expression stability of candidate internal reference genes. (**a**) Astaxanthin treatment groups. (**b**) Different tissues. (**c**) Different developmental stages.

The NormFinder algorithm estimates the stability of gene expression and ranks genes based on their stability. Genes with the least variation are considered the most stable. The results showed that among the 12 internal reference genes, their stability in different tissues ranked from high to low as follows: *RAB10* > *PFDN2* > *PABPC1* > *IDH3A* > *PAGR1* > *DLD* > *EEF1A* > *CYB5R3* > *MRPL17* > *PSMC3* > *MRPL43* > *NDUFS7* (Fig. 3b). For different developmental stages, the stability of the 12 internal reference genes ranked from high to low as follows: *NDUFS7* > *MRPL17* > *PAGR1* > *EEF1A* > *RAB10* > *MRPL43* > *PSMC3* > *IDH3A* > *DLD* > *PFDN2* > *PABPC1* > *CYB5R3* (Fig. 3c). In astaxanthin treatment groups, the 12 internal reference genes showed stability ranking from high to low as follows: *RAB10* > *PABPC1* > *PFDN2* > *DLD* > *IDH3A* > *PAGR1* > *MRPL43* > *MRPL17* > *PSMC3* > *NDUFS7* > *EEF1A* > *CYB5R3* (Fig. 3a).

#### GeNorm analysis

**Figure 4:**
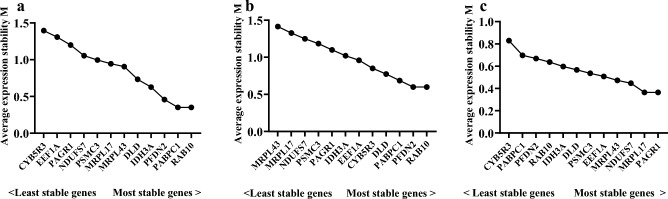
Analyze the M value of different internal reference bases. (**a**) Astaxanthin treatment groups. (**b**) Different tissues. (**c**) Different developmental stages.

GeNorm calculates the average degree of variation (M) to determine the stability of candidate reference genes. *RAB10* had the lowest M value, indicating it was the most stable in the astaxanthin treatment group and different tissues (Fig. 4a, b). *PAGR1* was the most stable, while *CYB5R3* was the least stable across different developmental stages (Fig. 4c).

**Figure 5:**
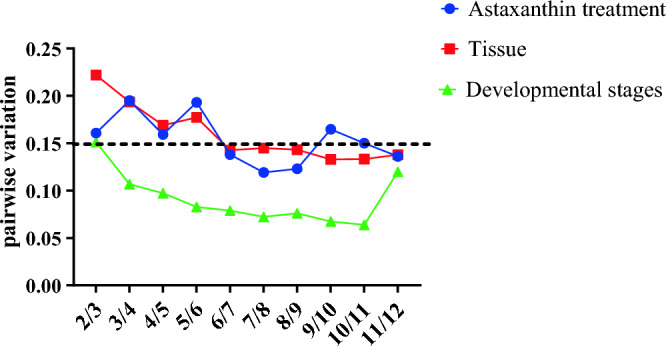
Determination of the optimal number of reference genes for normalization by geNorm. Blue color represents the astaxanthin treatment groups. Green represents different tissues. Red represents different developmental stages.

The V_n/n+1_ value determines the optimal number of internal reference genes for qRT-PCR experiments. If V_n/n+1_ < 0.15, n genes can be used as internal reference genes without the use of the n + 1th gene; if V_n/n+1_ > is 0.15, the screening should continue for n + 1 genes. In the astaxanthin treatment group, V_6/7_ < 0.15 (V_6/7_ = 0.14). Therefore, for reference gene selection in the astaxanthin treatment group, PABPC1 and RAB10 were the best combination of internal reference genes With V_6/7_ < 0.15 (V_6/7_ = 0.14), PFDN2 and RAB10 were the best combination of internal reference genes in different tissues of *L.* *erythropterus*. The minimum pairwise variability was V_3/4_ = 0.11, indicating that MRPL17 and PAGR1 were the most appropriate internal reference genes to be used simultaneously in different developmental stages of *L.* *erythropterus* (Fig. 5).

To improve the accuracy of the internal reference gene screen, we performed a comprehensive ranking of the 12 genes by RefFinder, an online tool that combines the four methods described above (See Supplementary Table S2). The comprehensive ranking showed that *RAB10* was the most stable candidate reference gene in different tissues and the astaxanthin treatment group of *L.* *erythropterus*. *NDUFS7* and *MRPL17* are the most reliable reference gene combinations for different developmental stages.

#### Validation of the stability of reference genes

**Figure 6:**
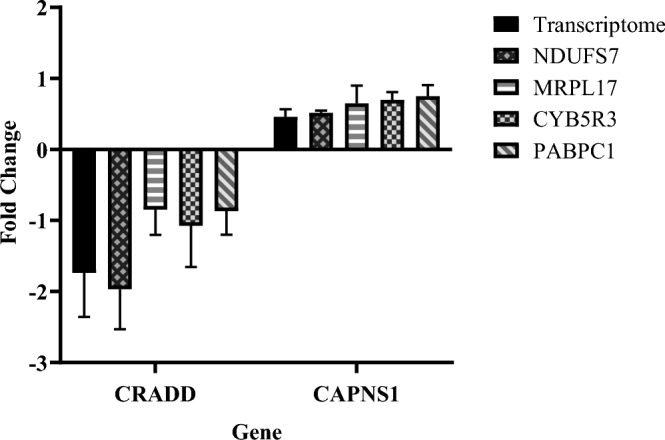
Different internal reference genes to analyse the relative expression levels of target genes in different developmental stages.

To further validate the accuracy of the results from the four analyses software, *NDUFS7*, *MRPL17*, *CYB5R3*, and *PABPC1* were used as internal reference genes to analyze the relative expression levels of the target genes *CRADD* and *CAPNS1* at 1 day and 20 days. Apoptosis is necessary for normal cell renewal and pattern reorganization during embryonic development. *CRADD* is an adapter protein containing a death domain that can oligomerize with *PIDD* and caspase-2 to initiate apoptosis^21^. The proteolytic activity of the calpain heterodimer impacts various cellular functions, including apoptosis, proliferation, migration, adhesion, and autophagy. The *CAPNS1* gene is a member of the calpain small subunit family, and studies have demonstrated its involvement in apoptosis functions^22^*.* The results showed in Fig. 6, the differential multiples obtained using *NDUFS7* as the internal reference gene are closer to the results of the transcriptomic analysis, indicating that *NDUFS7* is more stable than other internal references and is more suitable as an internal reference gene for different developmental stages of *L.* *erythropterus*.

## Discussion

Quantitative real-time PCR is commonly used for the study of gene expression. To minimize errors arising from both experimental and template factors, reference genes are typically selected for normalizing target gene expression^23^. The stability of reference gene expression is crucial during their selection process. Studies have shown that certain reference genes exhibit significant variability across different stages, tissues, cells, or experimental conditions^24–28^. For instance, in Hainan medaka tissue studies, ribosomal protein S4 (*RPS4*) and Elongation factor 1-beta (*EEF1B*) are recommended, while different embryonic developmental stages are better suited for ABC transporter (*MRP3*) and ribosomal protein S20 (*RPS20*)^24^. During ovarian development in *Paralichthys* *olivaceus*, actin beta (*ACTB*) and cathepsin D (*CTSD*) were found to be stably expressed in the hypothalamus, pituitary, ovary, and liver, respectively^25^. In rainbow trout exposed to cadmium and copper, stably expressed reference genes in gills were *HPRT* and *β-actin*, whereas *rpl8* and *EEF1a* showed stability in the skin^27^. Therefore, the selection of the best reference genes should be tailored to specific experimental conditions and sample types. Many scholars consider screening internal reference genes based on reads per kilobases per million reads (RPKM) in transcriptome databases as an efficient, rapid, and reliable method. For example, Yang et al. utilized transcriptome and genome sequence information for *Brassica* *napus* to identify 40 candidate endogenous genes. They subsequently verified the expression stability of these candidates using qRT-PCR, ultimately selecting GDP dissociation inhibitor protein (*GDI1*), triangular pentatricopeptide repeat protein (*PPR*), ubiquitin-based associated proteins (*UBA*), fat-soluble tea polyphenols (*OTP80*), and epsin n-terminal homology domain (*ENTH*) as the most suitable endogenous genes^29^. In this study, we used data from three different transcriptome databases from different developmental stages, different tissues, and astaxanthin-treated groups of *L.* *erythropterus*, and first filtered out the genes with unstable expression, and then took an intersection of genes with stable and high expression in all three, from which 12 candidate endogenous genes were selected based on functional gene annotations and BLAST results. This method allowed us to efficiently and accurately identify suitable endogenous genes for our research.

We assessed the stability of *L.* *erythropterus* reference genes by comparing results from four software analysis methods^17–20^. The stability rankings from geNorm, NormFinder, and the delta-Ct method were quite similar, especially concerning the most and least stable genes. However, the results from the different software packages were not entirely consistent. This discrepancy may be attributed to the fact that geNorm and NormFinder assess stability based on changes in the Ct value^18,20^, while BestKeeper calculates stability using the correlation coefficient of the Ct value^18^. We also used RefFinder, a simple and fast online software tool that integrates geNorm, NormFinder, BestKeeper, and the delta-Ct method. It calculates the geometric mean of their algorithms to screen and analyze reference genes^30^. To avoid potential bias associated with relying on a single program, this study combined results obtained from multiple programs to create a comprehensive ranking of the stability of candidate internal reference genes.

*RAB10* emerged as the most suitable gene when screened across various tissues and astaxanthin treatment groups. It is plausible to hypothesize that the administration of astaxanthin had no discernible impact on the endogenous screening of *L.* *erythropterus* tissues. *RAB10*, a member of the RAS superfamily of small GTPases, plays a pivotal role in catalyzing tyrosine phosphorylation of substrate proteins, thereby initiating a cascade of protein phosphorylation events that ultimately drive cellular proliferation^31^. In contrast, the *NDUFS7* gene exhibited the highest stability in expression across different developmental stages. *NDUFS7* is intricately linked with mitochondrial synthesis, as it encodes a crucial subunit within the mitochondrial respiratory chain complex^32^. This subunit is pivotal for the assembly of the respiratory chain complex within mitochondria, contributing to overall cellular energy metabolism. To further validate the accuracy of the software’s predictive results, this study selected *NDUFS7*, *MRPL17*, *CYB5R3*, and *PABPC1* as internal reference genes and examined the relative expression levels of two genes during different developmental stages. The experimental results further confirmed that the results for *NDUFS7* are closer to the transcriptomic data, indicating that it is a suitable internal reference gene for different developmental stages of *L.* *erythropterus*.

Generally, correction using a single reference gene is limited, so two or more stably expressed reference genes are needed to normalize target gene expression^3^. GeNorm software calculates the V value for paired variation to determine the optimal number of reference genes^33^, which is necessary in studies requiring precise expression quantification. Pairwise variability analysis in the GeNorm program showed that the optimal number of internal reference genes to be used in quantitative experiments was two in different tissues, at different developmental stages, and in the astaxanthin treatment groups. Therefore, using two reference genes can provide more accurate results when quantifying target gene expression.

## Conclusions

This is the first study to co-select candidate reference genes from three transcriptomes of *L.* *erythropterus* and then screen for suitable internal reference genes in different tissues, different developmental stages, and astaxanthin treatment groups conditions. According to the results of this study, *RAB10* and *PFDN2* exhibited relatively stable expression patterns across different tissue groups and astaxanthin treatment groups, while *NDUFS7* and *MRPL17* proved to be the most reliable reference gene combinations across various developmental stages. These identified internal reference genes can effectively normalize gene expression studies in *L.* *erythropterus* and may be useful for other similar fish under similar experimental conditions.

### Supplementary Information

Supplementary Information.

## Data Availability

Transcriptome data: NCBI accession numbers for different developmental periods PRJNA946949, NCBI accession numbers for astaxanthin-treated group PRJNA1039708, transcriptome data from different tissues was collected from our previous studies^15^.

## References

[1] Costa-Silva J, Domingues D, Lopes FM (2017). RNA-seq differential expression analysis: An extended review and a software tool. PLoS One.

[2] Kozera B, Rapacz M (2013). Reference genes in real-time PCR. J. Appl. Genet..

[3] Bustin SA (2009). The MIQE guidelines: Minimum information for publication of quantitative real-time PCR experiments. Clin. Chem..

[4] Su J, Zhang R, Dong J, Yang C (2011). Evaluation of internal control genes for qRT-PCR normalization in tissues and cell culture for antiviral studies of grass carp (*Ctenopharyngodon*
*idella*). Fish Shellfish Immunol..

[5] Fernandes JMO, Mommens M, Hagen Ø, Babiak I, Solberg C (2008). Selection of suitable reference genes for real-time PCR studies of Atlantic halibut development. Comp. Biochem. Physiol. Part B Biochem. Mol. Biol..

[6] Infante C (2008). Selection of housekeeping genes for gene expression studies in larvae from flatfish using real-time PCR. BMC Mol. Biol..

[7] Liao Z (2021). Screening of reference genes in tiger puffer (*Takifugu*
*rubripes*) across tissues and under different nutritional conditions. Fish Physiol. Biochem..

[8] Ryan MT, Collins CB, O’Doherty JV, Sweeney T (2010). Selection of stable reference genes for quantitative real-time PCR in porcine gastrointestinal tissues. Livest. Sci..

[9] Lin L, Li CH, Xu SN, Liu Y, Xiao YY (2015). Isolation and characterization of novel polymorphic microsatellite markers for *Lutjanus*
*erythropterus*. Genet. Mol. Res..

[10] Xu Z (2023). Weighted gene co-expression network analysis of red body color formation of crimson snapper, *Lutjanus*
*erythropterus*. Aquac. Rep..

[11] Barredo J, García-Estrada C, Kosalkova K, Barreiro C (2017). Biosynthesis of astaxanthin as a main carotenoid in the heterobasidiomycetous yeast *Xanthophyllomyces*
*dendrorhous*. J. Fungi.

[12] Yu W, Liu J (2020). Astaxanthin isomers: Selective distribution and isomerization in aquatic animals. Aquaculture.

[13] Begum H, Yusoff FMd, Banerjee S, Khatoon H, Shariff M (2016). Availability and utilization of pigments from microalgae. Crit. Rev. Food Sci. Nutr..

[14] Wang Z (2010). Performance of mitogenomic coding regions within genus Lutjanus molecular phylogenetics: Performance of mitogenomic coding regions within genus Lutjanus molecular phylogenetics. J. Fish. China.

[15] Liang Q (2022). Analysis of opsin gene family of crimson snapper (*Lutjanus*
*erythropterus*). Gene.

[16] Zhang Y-P (2015). Morphological characters and transcriptome profiles associated with black skin and red skin in crimson snapper (*Lutjanus*
*erythropterus*). IJMS.

[17] Vandesompele J, Preter KD, Roy NV, Paepe AD (2002). Accurate normalization of real-time quantitative RT-PCR data by geometric averaging of multiple internal control genes. Genome Biol..

[18] Pfaffl MW, Tichopad A, Prgomet C, Neuvians TP (2004). Determination of stable housekeeping genes, differentially regulated target genes and sample integrity: BestKeeper—Excel-based tool using pair-wise correlations. Biotechnol. Lett..

[19] Andersen CL, Jensen JL, Ørntoft TF (2004). Normalization of real-time quantitative reverse transcription-PCR data: A model-based variance estimation approach to identify genes suited for normalization, applied to bladder and colon cancer data sets. Cancer Res..

[20] Silver N, Best S, Jiang J, Thein SL (2006). Selection of housekeeping genes for gene expression studies in human reticulocytes using real-time PCR. BMC Mol. Biol..

[21] Di Donato N (2016). Mutations in CRADD result in reduced caspase-2-mediated neuronal apoptosis and cause megalencephaly with a rare lissencephaly variant. Am. J. Hum. Genet..

[22] Bertoli C, Copetti T, Lam EW-F, Demarchi F, Schneider C (2009). Calpain small-1 modulates Akt/FoxO3A signaling and apoptosis through PP2A. Oncogene.

[23] Chen Y (2015). Selection of reference genes for quantitative real-time PCR normalization in creeping bentgrass involved in four abiotic stresses. Plant Cell Rep..

[24] Dong Z (2019). Evaluation of reference genes for quantitative real-time PCR analysis of gene expression in Hainan medaka (*Oryzias*
*curvinotus*). Gene Rep..

[25] Gao Y, Gao Y, Huang B, Meng Z, Jia Y (2020). Reference gene validation for quantification of gene expression during ovarian development of turbot (*Scophthalmus*
*maximus*). Sci. Rep..

[26] Lang X, Wang L, Zhang Z (2016). Stability evaluation of reference genes for real-time PCR in zebrafish (*Danio*
*rerio*) exposed to cadmium chloride and subsequently infected by bacteria *Aeromonas*
*hydrophila*. Aquat. Toxicol..

[27] Shekh K, Tang S, Niyogi S, Hecker M (2017). Expression stability and selection of optimal reference genes for gene expression normalization in early life stage rainbow trout exposed to cadmium and copper. Aquat. Toxicol..

[28] Zhou Q (2020). De novo sequencing and chromosomal-scale genome assembly of leopard coral grouper, *Plectropomus*
*leopardus*. Mol. Ecol. Resour..

[29] Yang H (2014). Selection and evaluation of novel reference genes for quantitative reverse transcription PCR (qRT-PCR) based on genome and transcriptome data in *Brassica*
*napus* L. Gene.

[30] Xie F, Xiao P, Chen D, Xu L, Zhang B (2012). miRDeepFinder: A miRNA analysis tool for deep sequencing of plant small RNAs. Plant Mol. Biol..

[31] Steger M (2016). Phosphoproteomics reveals that Parkinson’s disease kinase LRRK2 regulates a subset of Rab GTPases. eLife.

[32] Kania E (2023). LRRK2 phosphorylation status and kinase activity regulate (macro)autophagy in a Rab8a/Rab10-dependent manner. Cell Death Dis..

[33] Kidd M (2007). GeneChip, geNorm, and gastrointestinal tumors: Novel reference genes for real-time PCR. Physiol. Genom..

