# Supplementary material for: Systematic selection of suitable reference genes for quantitative real-time PCR normalization studies of gene expression in Lutjanus erythropterus
Source: Sci Rep. 2024 Jun 10;14:13323. doi: 10.1038/s41598-024-63335-x (PMC11164968; doi:10.1038/s41598-024-63335-x)
Supplement: Supplementary file 1 — Supplementary Information. [file 41598_2024_63335_MOESM1_ESM.pdf]

**Supplementary Figure 1.** Standard curves for the 12 candidate reference genes

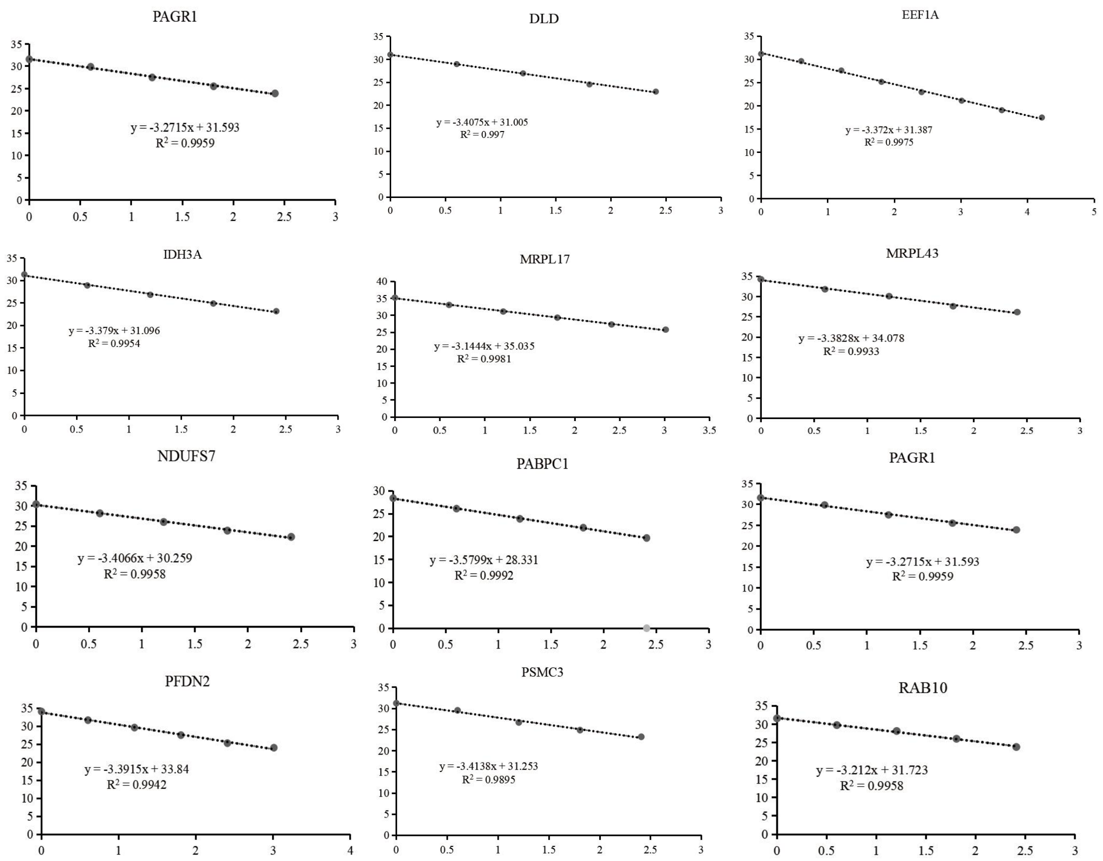


**Supplementary Figure 2.** Solubilization curves for 12 candidate reference genes

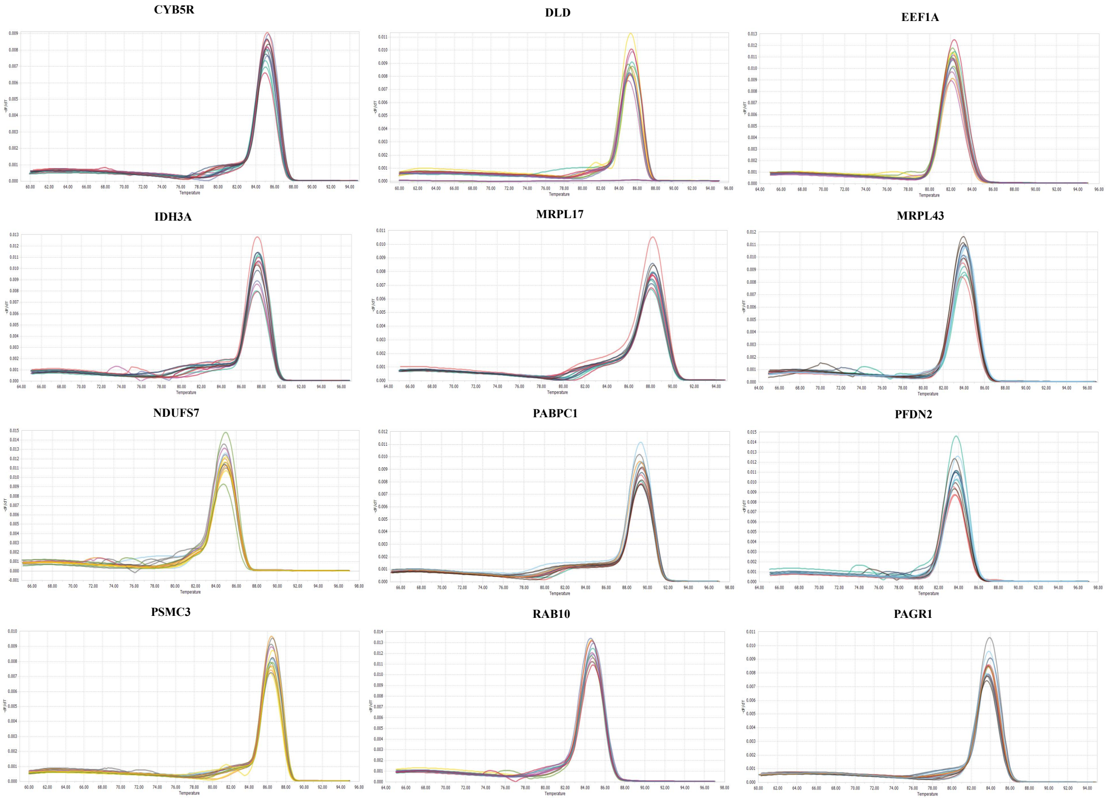

**Supplementary table 1.** Grunnleggende informasjon

| Primer name | Primer sequence (5'-3') |
|---|---|
| *CRADD* | F: TGACTCCATCGTTCCGTTTC |
| | R: GCAGGAAGGCGTCAAAGG |
| *CAPNS1* | F: AGACCTTTCCAAGAAGAGCCC |
| | R: AGGCACTGTTGGTTGTGGATAC |

**Supplementary table 2.** Ranking of candidate reference genes by geNorm, NormFinder, BestKeeper, comparative ΔCt method, and overall rank

| | Delta CT | BestKeeper | Normfinder | Genorm | Re fFinder |
|---|---|---|---|---|---|
| Astaxanthin treatment groups | RAB10 | PAGR1 | RAB10 | PABPC1 | RAB10 |
| | PFDN2 | DLD | PFDN2 | RAB10 | PABPC1 |
| | PABPC1 | RAB10 | PABPC1 | PFDN2 | PFDN2 |
| | DLD | PABPC1 | DLD | IDH3A | DLD |
| | IDH3A | PFDN2 | IDH3A | DLD | IDH3A |
| | MRPL43 | EEF1A | MRPL43 | MRPL43 | PAGR1 |
| | MRPL17 | MRPL43 | MRPL17 | MRPL17 | MRPL43 |
| | NDUFS7 | MRPL17 | NDUFS7 | PSMC3 | MRPL17 |
| | PSMC3 | IDH3A | PSMC3 | NDUFS7 | PSMC3 |
| | PAGR1 | PSMC3 | PAGR1 | PAGR1 | NDUFS7 |
| | EEF1A | CYB5R3 | EEF1A | EEF1A | EEF1A |
| | CYB5R3 | NDUFS7 | CYB5R3 | CYB5R3 | CYB5R |
| Different tissues | RAB10 | PAGR1 | RAB10 | PFDN2 | RAB10 |
| | PFDN2 | IDH3A | PABPC1 | RAB10 | PFDN2 |
| | PABPC1 | EEF1A | PFDN2 | PABPC1 | PABPC1 |
| | DLD | PABPC1 | DLD | DLD | IDH3A |
| | IDH3A | PFDN2 | IDH3A | CYB5R3 | PAGR1 |
| | EEF1A | MRPL17 | EEF1A | EEF1A | DLD |
| | CYB5R3 | MRPL43 | PAGR1 | IDH3A | EEF1A |
| | PAGR1 | RAB10 | CYB5R3 | PAGR1 | CYB5R3 |
| | PSMC3 | DLD | PSMC3 | PSMC3 | MRPL17 |
| | MRPL17 | PSMC3 | MRPL17 | NDUFS7 | PSMC3 |
| | NDUFS7 | CYB5R3 | NDUFS7 | MRPL17 | MRPL43 |
| | MRPL43 | NDUFS7 | MRPL43 | MRPL43 | NDUFS7 |
| Different developmental stages | NDUFS7 | RAB10 | NDUFS7 | MRPL17 | NDUFS7 |
| | MRPL17 | EEF1A | MRPL17 | PAGR1 | MRPL17 |
| | EEF1A | NDUFS7 | EEF1A | NDUFS7 | PAGR1 |
| | PAGR1 | PAGR1 | PAGR1 | MRPL43 | EEF1A |
| | MRPL43 | MRPL17 | MRPL43 | EEF1A | RAB10 |

| | | | | |
|---|---|---|---|---|
| PSMC3 | PFDN2 | IDH3A | PSMC3 | MRPL43 |
| IDH3A | PABPC1 | PSMC3 | DLD | PSMC3 |
| DLD | IDH3A | DLD | IDH3A | IDH3A |
| RAB10 | PSMC3 | RAB10 | RAB10 | DLD |
| PFDN2 | MRPL43 | PFDN2 | PFDN2 | PFDN2 |
| PABPC1 | DLD | PABPC1 | PABPC1 | PABPC1 |
| CYB5R3 | CYB5R3 | CYB5R3 | CYB5R3 | CYB5R3 |
